# Intermolecular vs molecule–substrate interactions: A combined STM and theoretical study of supramolecular phases on graphene/Ru(0001)

**DOI:** 10.3762/bjnano.2.42

**Published:** 2011-07-12

**Authors:** Michael Roos, Benedikt Uhl, Daniela Künzel, Harry E Hoster, Axel Groß, R Jürgen Behm

**Affiliations:** 1 Institute of Surface Chemistry and Catalysis, Ulm University, D-89069 Ulm, Germany; 2 Institute of Theoretical Chemistry, Ulm University, D-89069 Ulm, Germany

**Keywords:** graphene film, intermolecular interaction, large organic molecules, substrate–adsorbate interaction, supramolecular structure

## Abstract

The competition between intermolecular interactions and long-range lateral variations in the substrate–adsorbate interaction was studied by scanning tunnelling microscopy (STM) and force field based calculations, by comparing the phase formation of (sub-) monolayers of the organic molecules (i) 2-phenyl-4,6-bis(6-(pyridin-3-yl)-4-(pyridin-3-yl)pyridin-2-yl)pyrimidine (3,3'-BTP) and (ii) 3,4,9,10-perylene tetracarboxylic-dianhydride (PTCDA) on graphene/Ru(0001). For PTCDA adsorption, a 2D adlayer phase was formed, which extended over large areas, while for 3,3'-BTP adsorption linear or ring like structures were formed, which exclusively populated the areas between the maxima of the moiré structure of the buckled graphene layer. The consequences for the competing intermolecular interactions and corrugation in the adsorption potential are discussed and compared with the theoretical results.

## Introduction

It is well known that the formation of highly ordered 2D supramolecular networks on smooth surfaces, such as metal substrates or highly oriented pyrolytic graphite (HOPG), is mainly governed by the intermolecular interactions between adjacent molecules due to hydrogen bonding [1–4], covalent bonding [5–6], or, in the case of metal organic networks, by metal–ligand interactions [1,7–9]. In these cases, the interactions between the adlayer and the substrate, or more specifically, the local variations in that interaction, play a minor role. These interactions mainly determine the orientation of the resulting supramolecular structure with respect to the underlying substrate lattice [3,6,10–14]. This is mainly due to the fact that lateral variations in the interaction between the surface and a single bonding center in the admolecule, arising from the atomic structure of the surface, largely average out because of the imperfect match of the different bonding centers in the adsorbed molecule and the surface lattice. The situation is distinctly different if the adsorption potential is corrugated on a much larger length scale, exceeding that of the size of these admolecules, which is typically in the order of a few nanometers. Such long-range corrugations, however, are rather rare.

**Figure 1:**
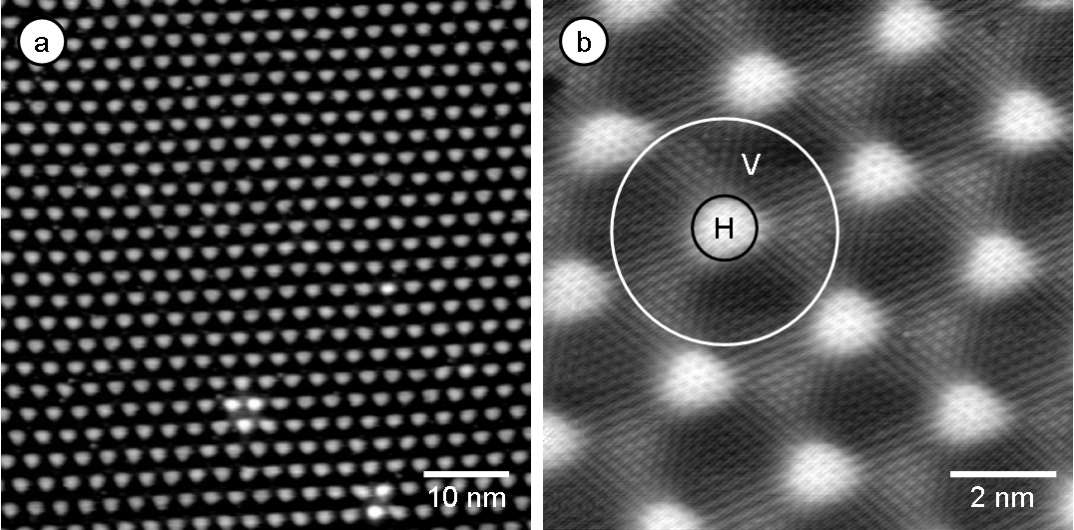
(a) Defect free graphene/Ru(0001) surface with typical moiré superstructure (*U*_T_ = −1.30 V, *I*_T_ = 60 pA, *T* = 130 K, 62 nm × 62 nm). (b) Atomically resolved STM image of the graphene/Ru(0001) surface (*U*_T_ = 0 V, *I*_T_ = 178 pA, T = 300 K, 10 nm × 10 nm) with different superimposed adsorption areas (H) = hill, (V) = valley.

One example of this effect is in metal supported graphene films. In the case of Ru as a support, it is known from LEED structure analysis and theory that the lattice mismatch between the graphene layer and the underlying metal substrate results in a buckling of the graphene layer and therefore in a distinct height corrugation of 1.5 Å [15–16]. The graphene/Ru(0001) surface is also known to exhibit a lateral corrugation of 30 Å, which is comparable with the outer dimensions of the molecules used in this study. Representative images of the graphene/Ru(0001) surface presented in Figure 1 underline the highly ordered long-range periodicity of the graphene adlayer with its characteristic moiré pattern (Figure 1a) and resolve the atomic structure in the high resolution image (Figure 1b). In the latter image, two different areas are marked, denoting the positions on top of the maxima of the moiré lattice of the graphene film (“hill positions” - H) and the lower parts between the maxima (“valley positions” - V). These are taken as representative for the different adsorption sites on the graphene/Ru(0001) surface.

Recently, we demonstrated, for the adsorption of 2-phenyl-4,6-bis(6-(pyridin-2-yl)-4-(pyridin-4-yl)pyridin-2-yl)pyrimidine (2,4’-BTP) molecules on a Ru(0001) supported graphene film, that such surfaces indeed exhibit not only a height corrugation but also a distinct corrugation in the adsorption potential [17]. This was explained by a mechanism in which the variation in the adsorption potential exceeds the intermolecular interactions, which favors population of the energetically favorable valley as compared to the formation of a 2D adlayer phase covering the entire surface, or islands of that phase covering part of the surface. Comparable structures were reported for graphene/Rh(111) [18]. Pronounced lateral variations in the adsorption potential on metal supported graphene monolayer films were previously reported also for metal deposition on such surfaces, e.g., for Ir on graphene/Ir(111) [19–20] or for Pt on graphene/Ru(0001) [21–23].

**Figure 2:**
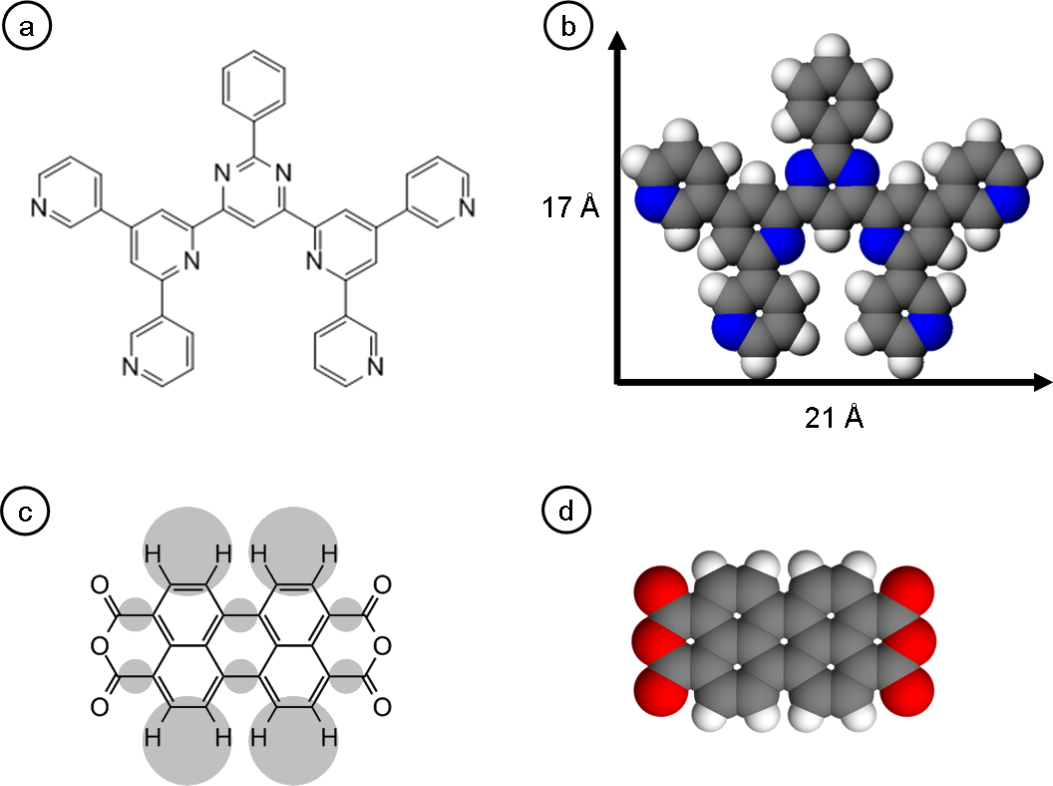
(a) Schematic and (b) space filling models with lateral dimensions of 3,3'-BTP. (c) Schematics and correlation between the STM image and the intermolecular structure of PTCDA, and (d) the space filling model of PTCDA.

In this paper we extend this study, by comparing the adsorption behavior of two different types of molecular systems with distinctly different intermolecular interactions, namely (i) 2-phenyl-4,6-bis(6-(pyridin-3-yl)-4-(pyridin-3-yl)pyridin-2-yl)pyrimidine (3,3'-BTP) [24–25] and (ii) 3,4,9,10-perylene tetracarboxylic dianhydride (PTCDA) on graphene/Ru(0001). Schematic representations and space filling models of these molecules are presented in Figure 2. For both molecules, the intermolecular interactions are dominated by hydrogen bonds. In the case of the 3,3'-BTP the numbers 3,3' indicate the position of the outer nitrogen atoms which are responsible for hydrogen bonding between adjacent molecules. The strengths of these hydrogen bonds, however, are rather different for the two molecules. In the case of the 3,3'-BTP molecules, weak C–H^…^N-type hydrogen bonds are formed which are typically in the range of 100 meV [25]. The C–H^…^O-type hydrogen bonds formed between PTCDA molecules are somewhat stronger and result in intermolecular interactions in the range of 130 meV [26].

In the following, we first present STM observations of the adlayer phases formed by these molecules on graphene/Ru(0001) and compare these with adlayers of the corresponding phases on HOPG as a model for a low corrugation substrate. Subsequently, we present results of theoretical considerations, including force field based calculations of the interaction between the graphene/Ru(0001) substrate and adsorbed molecules, and discuss the consequences for the adlayer phase formation.

## Results and Discussion

### STM imaging

**Figure 3:**
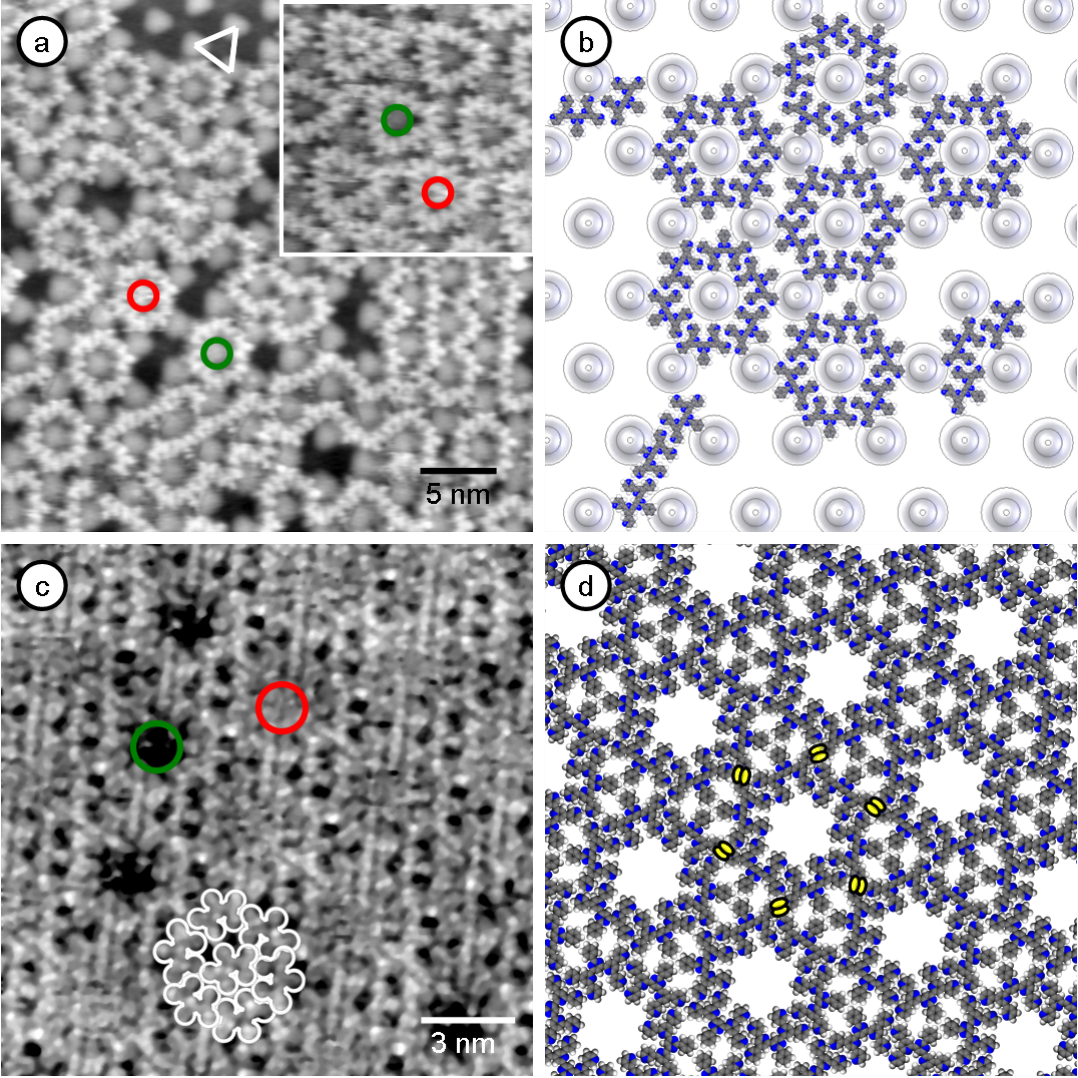
(a) STM image of 3,3'-BTP molecules on graphene (*U*_T_ = −2.36 V, *I*_T_ = 30 pA, *T* = 115 K, 35 nm × 35 nm). The inset shows the triangular structure with randomly filled (red) and unfilled (green) cavities (*U*_T_ = −2.36 V, *I*_T_ = 30 pA, *T* = 115 K, 9 nm × 9 nm). (b) True to scale model of 3,3'-BTP molecules on graphene illustrates the steric hindrance. (c) 3,3'-BTP on HOPG (*U*_T_ = −1.20 V, *I*_T_ = 44.7 pA, *T* = 300 K, 17 nm × 17 nm); The same hydrogen bonding configuration and hexagonal network, but without spacing between the hexagons. (d) Model of 3,3'-BTP on HOPG. Each molecule shares two hydrogen bonds with its adjacent hexagon (yellow ellipses).

Figure 3a shows an exemplary STM image of a low coverage 3,3'-BTP adlayer on graphene/Ru(0001), with sub-molecular resolution. We can clearly identify the molecules and the hexagonally arranged hills of the graphene adlayer (see marked triangle). The molecules are exclusively adsorbed in the valleys of the graphene film, while the hills remain unoccupied. This limitation to specific adsorption sites in combination with the distinct positions of the hydrogen bond donors and acceptors within the molecule results in the formation of 1D chain structures (Figure 3b), similar to findings recently reported for the adsorption of PTCDI molecules on graphene/Rh(111) [18].

Due to the position of the N atoms and of the resulting hydrogen bonds between the 3,3'-BTP molecules, the attachment of an additional molecule at the end of a molecular chain is not restricted to an anti-parallel arrangement, but can occur also at an angle of 60° with respect to the last molecule in the chain. The former is responsible for linear chain structures, the latter will lead to curved chain-like structures or hexagonal and triangular ring structures. Such structures consist mostly of six molecules and are formed around a hill (Figure 3a and Figure 3b). The inner diameter of these units leaves enough space for an additional molecule on top of the enclosed hill, which in some cases are trapped on these sites. These additional molecules seem to be freely rotating, as evidenced by their diffuse shape. Figure 3b shows the different possible adsorption geometries. The long range order, however, is rather poor, with only relatively small units and chain-like arrangements. The hills of the graphene act as spacers between individual molecular units. Due to the resulting separation of approximately 10 Å between adjacent units, intermolecular interactions between them can be neglected. The fact that the hills remain unoccupied for 3,3'-BTP on graphene/Ru(0001) indicates that these sites are significantly less favorable than the valley sites, i.e., the corrugation of the adsorption potential exceeds possible additional intermolecular interactions between the different units.

Adsorption of 3,3'-BTP-molecules on HOPG led to networks with similar types of building blocks as formed on graphene/Ru(0001). At both the solid–liquid and the solid–gas interface we predominantly found a chiral 2D hexagonal network consisting of ring-like units of six molecules (Figure 3d) [24]. At the solid–gas interface, the resulting hexagonal network seems to be the most stable phase, as indicated by its coexistence with a 2D gas at lower coverage. Again, these rings provide enough space for adsorption of an additional central molecule. In this network, the hexagonal units are interconnected, i.e., there are not only interactions between the molecules within the hexagons, but also between molecules of adjacent hexagons (marked yellow in Figure 3d). This is in contrast to the clear separation between the rings on graphene/Ru(0001). In addition to the 2D hexagonal network, several 2D phases consisting of linear arrangements of 3,3'-BTP molecules were observed on HOPG [24]. These linear networks are characterized by their anti-parallel arrangement of the molecules. Similar to the adsorption of 2,4’-BTP molecules [4,12] on Ag thin films, these linear networks also exhibit intermolecular bonds between the linear elements. Finally, it is interesting to note that comparable 2D phases based on bent structures with 60° angles between different linear parts were not observed on HOPG. Molecular models show that in such phases hydrogen bonds are not possible between adjacent strings, leaving them energetically less favorable compared to phases based on linear structures.

**Figure 4:**
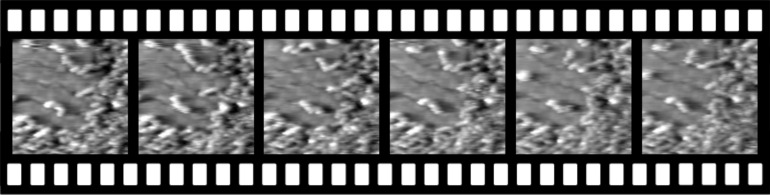
Sequence of time resolved images recorded at 2 fps showing every 10th image (time between single frames 5 s). Graphene/Ru(0001) partly covered with 3,3'-BTP molecules.

The influence of thermal activation on the structure formation is illustrated in a series of snapshots recorded at 2 frames per second (fps, every 10th image shown) at room temperature with a home built video-STM (Figure 4). Upon imaging at higher tunnel current (smaller tip–sample distance), tip induced removal of 3,3'-BTP molecules from a fully covered graphene/Ru(0001) surface led to a local molecule-free surface area. Switching back to normal tunnelling conditions, 3,3'-BTP molecules diffused in from the surrounding area and re-populated this area. This re-occupation proceeds via growth of chain-like aggregates of the molecules, which follow the valley structure of the graphene while the hills remain unoccupied. Hence, even at room temperature thermal activation is not sufficient for growing over the graphene hills, reflecting the pronounced lateral corrugation of the 3,3'-BTP adsorption potential on the graphene/Ru(0001) surface.

**Figure 5:**
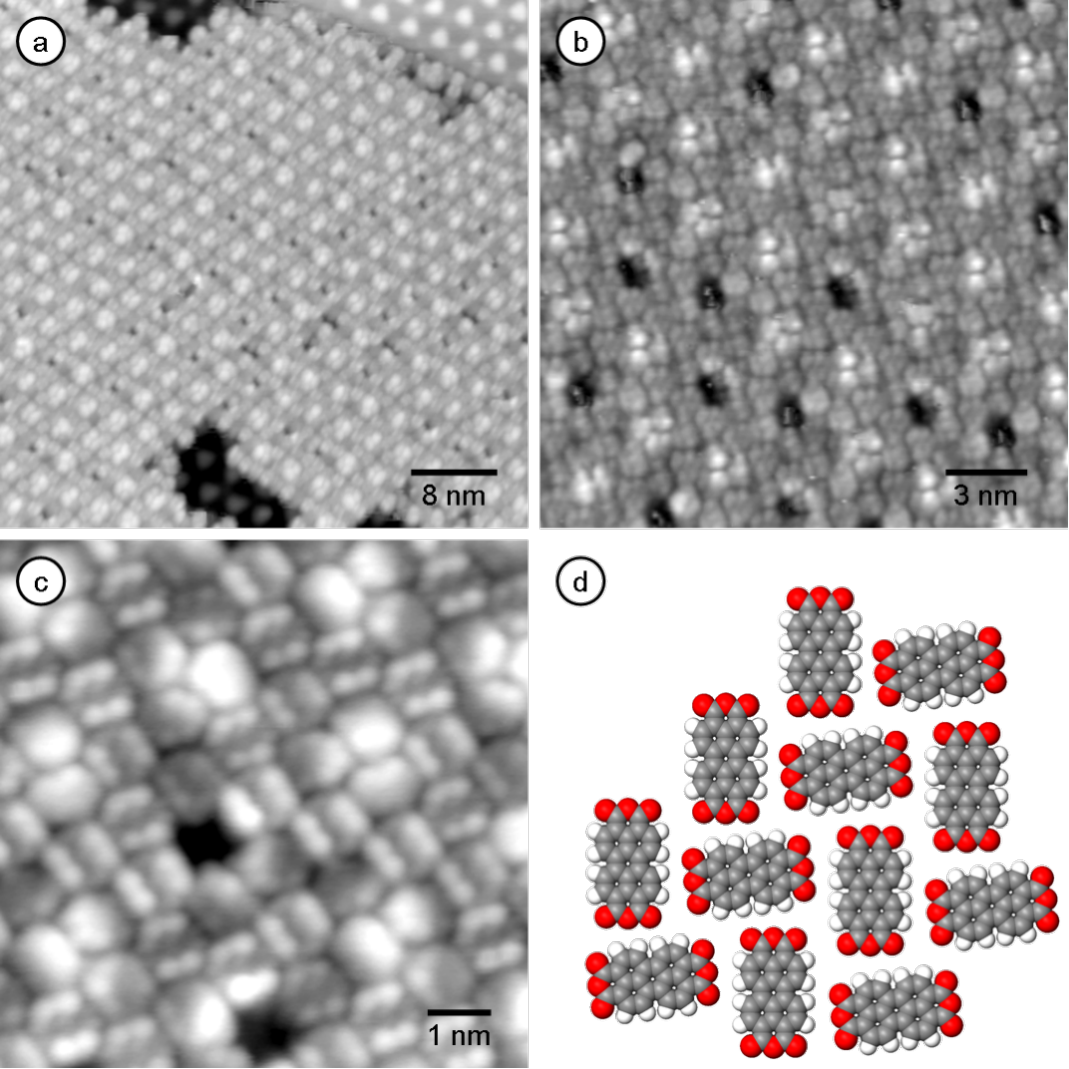
(a) Sub-monolayer of PTCDA on graphene/Ru(0001) (*U*_T_ = −0.61 V, *I*_T_ = 180 pA, *T* = 125 K, 49 nm × 49 nm) with simultaneous imaging of the underlying moiré of the graphene layer. (b) Not all hills are covered with PTCDA molecules. Fuzzy impression of molecules on hills indicative of (frustrated) rotation (*U*_T_ = −0.88 V, *I*_T_ = 50 pA, *T* = 125 K, 19 nm × 19 nm). (c) Sub-molecular resolution image revealing the adsorption geometry of the PTCDA molecules. (8 nm × 8 nm, *U*_T_ = 0.97 V, *I*_T_ = 110 pA, *T* = 125 K) (d) Model of the adsorption geometry of PTCDA molecules.

Figure 5 shows the network formation of PTCDA molecules on graphene/Ru(0001). Even in the sub-monolayer regime, the molecules arrange in a periodic herringbone-like arrangement (Figure 5), similar to the phases described for other substrates such as HOPG [27–29] and various metal substrates such as Au(111) [30–34], Ag(110) [35] or Cu(110) [36]. In contrast to the weakly interacting BTP molecules, the PTCDA adlayer covers the entire surface, i.e., the PTCDA molecules follow the up and down of the moiré superstructure of the graphene/Ru(0001) layer (Figure 5a). Since the unit cell of the herringbone structure does not fit perfectly to the periodicity of the underlying graphene/Ru(0001) substrate, an additional long-range structural modulation is obtained. The image in Figure 5a resolves both the PTCDA molecular adlayer and, in a hole of the adlayer, the underlying moiré structure with its hills and valleys. While the majority of the hills are covered by the PTCDA adlayer, some also result in local defects within the network and produce distinct voids of about the size of one molecule (Figure 5a–c). These voids indicate that the graphene/Ru(0001) substrate also exhibits a significant corrugation in the adsorption potential for PTCDA, although this is less pronounced, relative to intermolecular interactions, than for 3,3'-BTP. Figure 5c shows a sub-molecular resolution image of PTCDA on graphene/Ru(0001). Comparison with the model of a single PTCDA molecule in Figure 2c reveals the same sub-molecular structural details within the molecule. Similar structural results have recently been reported for the adsorption of PTCDA on SiC(0001) where the PTCDA film is grown over the corrugated surface without any disturbance due to the different adsorption sites [37].

### Calculations

To determine the corrugation of the adsorption potential, we summed up the different contributions to the adsorption energy (molecule–graphene and molecule–Ru interaction) and subtracted the adsorption energy of a “hill” site from that of a “valley” position. Table 1 and Table 2 show the differences between these sites for different force fields. Clearly, the calculated binding energies strongly depend on the force field used, as found before in force field calculations addressing 3,3'-BTP adsorption on graphite [38]. Nevertheless, although the absolute values may vary with the applied force field, the differences between binding energies at the two sites, reflecting the lateral corrugation of the potential energy of the substrate, are reasonably close, both for 3,3'-BTP and PTCDA.

**Table 1:** Adsorption energies for 3,3'-BTP on graphene for different adsorption sites and corrugation Δ*E* in eV.

	hill	valley	Δ*E*

Compass	−3.346	−3.971	−0.625
CVFF	−6.120	−7.105	−0.985
Dreiding, Gasteiger	−3.400	−4.093	−0.693
Dreiding, QEq	−3.388	−4.013	−0.625
UFF, Gasteiger	−3.889	−4.669	−0.780
UFF, QEq	−3.853	−4.538	−0.685

**Table 2:** Adsorption energies of PTCDA for different adsorption sites and corrugation Δ*E* in eV.

	hill	valley	Δ*E*

Compass	−1.889	−2.324	−0.435
CVFF	−3.405	−4.095	−0.690
Dreiding, Gasteiger	−2.417	−2.875	−0.458
Dreiding, QEq	−3.037	−3.587	−0.550
UFF, Gasteiger	−2.248	−2.739	−0.491
UFF, QEq	−2.600	−3.171	−0.571

The corrugation of the adsorption energy can be compared with the intermolecular interactions. For the adsorption of 3,3'-BTP molecules, the STM images shown above reveal that very similar hexagonal local units are formed upon adsorption on HOPG and on graphene/Ru(0001). On both surfaces, these units consist of six molecules in a hexagonal arrangement. The main difference lies in the spacing between the different units. In the case of graphene/Ru(0001), they are separated by the hills of the graphene. On HOPG, in contrast, they are interconnected and therefore additionally stabilized by hydrogen bonds between adjacent molecules of neighboring units. The additional double hydrogen bonds are in the range of 0.14 eV per double bond [25]. Therefore the additional stabilization of a single molecule within a unit is only 0.07 eV per molecule (half of the double bond). Comparing this value with the potential energy corrugation, which depending on the applied force field varies between 0.625 and 0.985 eV per molecule (Table 1), we see that these additional intermolecular interactions which apply for higher adsorbate densities would be much lower than the corrugation of the adsorption potential. Hence, at sub-monolayer coverage it is energetically preferable to adsorb only in valley sites and their connections, rather than to form islands of 2D interconnected networks which would also occupy hill sites. Therefore, the tendency to avoid the hills can be easily rationalized by comparing the intermolecular interactions and the corrugation in the 3,3'-BTP-substrate interactions.

**Figure 6:**
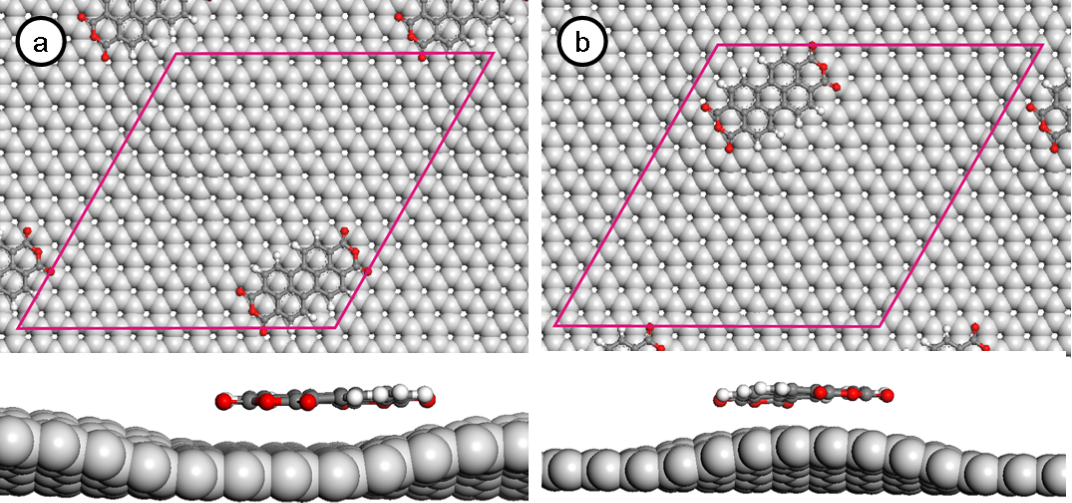
Optimized structure of PTCDA in (a) the valley position and (b) the hill position.

The same procedure was applied for PTCDA molecules adsorbed on graphene/Ru(0001). Figure 6 shows the adsorption geometry for a single PTCDA molecule (a) on a valley and (b) on a hill position, with top and side view of the adsorption geometry. Table 2 shows the calculated adsorption energy for both the hill and the valley position and the resulting corrugation of the adsorption potential ΔE for PTCDA molecules on graphene/Ru(0001) for different force fields. Dependent on the applied force field, the resulting Δ*E* ranges from −0.435 to −0.690 eV. To rationalize the different behavior of the PTCDA molecules, we again compare the corrugation of the adsorption energy with the intermolecular interaction between adjacent molecules in the herringbone configuration of PTCDA. Recent DFT calculations by Mura et al. [26] have shown that the stabilization energy per molecule is between −0.585 and −0.67 eV per molecule, depending on the exact herringbone structure [26]. Comparing these values with the values for the corrugation of the adsorption potential between the different adsorption states (see above) reveals that the additional intermolecular stabilization energies for the PTCDA molecules (over)compensate for the corrugation within the adsorption potential. Hence in this case, formation of islands of a 2D network is energetically favorable compared to a phase with 1D strings between the hills or ring units around the hills of the graphene/Ru(0001) substrate, in perfect agreement with the experimental findings.

These results illustrate the detail of microscopic understanding that can be extracted from combined STM experiments and state-of-the-art calculations. They also support the validity of structural concepts for the self assembly of supramolecular networks for cases where the adsorption potential on the substrate is highly corrugated, instead of the normal ‘smooth’ substrates.

## Conclusion

We have shown by STM imaging that (i) there are distinct differences in the adlayer structures of 3,3'-BTP on HOPG compared to 3,3'-BTP on graphene/Ru(0001), while (ii) for PTCDA, similar structures are formed on both substrates. In the first case, the adlayer forms 1D chains around the hills of the graphene/Ru(0001), but a 2D interconnected network on HOPG, while in the second case 2D networks are formed on both substrates. Qualitatively, these differences can be explained by the competition between intermolecular interactions and the lateral variation of the adsorption potential, i.e., of the molecule–substrate interactions. In the case of the 3,3'-BTP molecules, the intermolecular interactions are significantly weaker than the potential energy corrugation of the surface, rendering the occupation of hill sites, and hence the formation of 2D networks, energetically unfavorable. In the case of PTCDA molecules, the lateral corrugation of the adsorption potential must be overcompensated by stronger intermolecular interactions. These ideas are fully supported on a quantitative scale by a combination of force field and density functional theory based calculations, which reveal much stronger intermolecular interactions for PTCDA than for 3,3'-BTP, while the difference in binding energy on valley sites (favorable) and on hill sites (unfavorable), and hence the lateral corrugation of the adsorption potential on the graphene/Ru(0001) substrate, is of similar magnitude for both molecules.

## Experimental

### Experiments

The experiments were performed in a standard ultrahigh vacuum (UHV) system (base pressure 2 × 10^−10^ mbar), equipped with a commercial variable temperature scanning tunneling microscope (STM) (Specs, STM 150 “Aarhus”) and facilities for sample preparation, such as an Ar^+^ ion sputter gun and evaporation sources for the deposition of organic molecules.

A Ru(0001) crystal (Mateck) was cleaned by standard procedures, including 3–4 cycles of Ar^+^ ion sputtering (0.5 kV Ar^+^ ions, 5 μA cm^−2^, 15 min), followed by flash annealing to 1650 K. Remaining carbon impurities were removed by oxidation, involving oxygen adsorption (10 L O_2_: 1 L = 1.33 × 10^−6^ mbar s^−1^) and subsequent repeated flash annealing to 1650 K. The freshly prepared surface exhibited 50–200 nm wide, atomically flat terraces, separated by monolayer steps. The graphene layer was prepared by exposing the Ru(0001) surface to ethylene for prolonged time at elevated temperatures (5 × 10^−9^ mbar ethylene, 2 h at 1000 K). The structural quality of the graphene layer was checked by STM in constant current mode at temperatures of around 100 K.

3,3'-BTP molecules (provided by U. Ziener, Ulm University) were deposited from a home-built, resistively heated, Knudsen cell at 583 K. The PTCDA molecules (Merck, 98% purity) were evaporated from a commercial evaporation source (Ventiotec, OVD-3) at 628 K. Prior to evaporation, the PTCDA was cleaned in UHV by a temperature gradient sublimation technique using a resistively heated quartz tube. After deposition the sample was annealed to 583 K to improve the structural quality of the molecular film.

### Theoretical Methods

To complement and rationalize the experimental findings above, force field calculations were performed to determine the site specific adsorption energies for 3,3'-BTP and PTCDA on graphene/Ru(0001). Due to the large size of the system, quantum chemical calculations were too computationally expensive. The surface was modelled with three layers of Ru and one layer of graphene on top, using a commensurate lattice with a (12 × 12) graphene unit cell on a (11 × 11) Ru(0001) cell with a lattice constant of 29.96 Å, in agreement with experiment, using the coordinates obtained in recent density functional theory calculations from a combined experimental and theoretical study [39]. These surfaces coordinates were then kept fixed in the subsequent relaxation of adsorbed PTCDA and BTP molecules. The interactions between molecule–graphene and molecule–Ru were treated as being additive. For the modelling of the molecule–graphene interactions, a single molecule was placed on top of the two different adsorption sites (“hill” and “valley”) and four different force fields were used to optimize the adsorption geometry of the adsorbate (Compass [40], CVFF [41], Dreiding [42], and UFF [43] as implemented in the Accelrys Materials Studio program package). Note that the “hill” position does not correspond to a true local minimum, so the structure optimization was performed for the internal molecular degrees of freedom with the center of mass of the molecule being on top of the hill. For Dreiding and UFF, we applied both the Gasteiger [44] and the QEq charging method [45]. For a reliable description of the interactions between the molecules and the ruthenium surface, force fields are normally not well suited since they do not accurately reproduce metallic properties. Instead, we used a semi-empirical dispersion correction scheme [46–47], which was originally used for the inclusion of van der Waals interactions in standard DFT calculations. These two contributions (adsorbate–graphene and adsorbate–metal) were then added in order to obtain total adsorption energies of the molecules on the graphene/Ru(0001) substrate.
